# How addiction fuels innovation: a mixed-methods study on the psychological trade-offs of digital labor in gig economy

**DOI:** 10.3389/fpsyg.2025.1701187

**Published:** 2025-12-16

**Authors:** Bingnan Liu, Vincenzo Liu, Kai He, Jiayu Ou

**Affiliations:** 1 School of Business, Macau University of Science and Technology, Taipa, Macao SAR, China; 2 International Business School, Jinan University, Zhuhai, China; 3 School of Finance, Zhongnan University of Economics and Law, Wuhan, China

**Keywords:** digital labor psychology, crowdworkers, innovation, addiction, gamification affordances, mixed method

## Abstract

As digitalization reshapes the global labor market, digital laborers in the gig economy face distinct psychological pressures stemming from constant hyperconnectivity, algorithm-driven coordination, and inherently unstable employment relationships. Employing a mixed-methods approach that combines data from 20 semi-structured interviews and 1,041 questionnaire responses, this study develops a research model to understand the mechanisms of digital crowdworkers’ addiction and its impact on enhancing innovation implementation. The results indicate that the affordance of gamification on crowdsourcing platforms, the self-worth pursuit of crowdsourced digital laborers, and their skill enhancement exert a positive impact on crowdworkers’ addiction and innovative behaviors. The findings suggest that digital crowdworkers’ behavioral addiction may motivate their innovative behavior, demonstrating that irrational psychological mechanisms can be transformed into instrumental innovation. The study reveals the dual effects of the platform economy on addictive behaviors and offers a new framework for organizations to balance short-term innovation efficiency gains with long-term sustainable development.

## 1 Introduction

With the deep penetration of digital technology across various sectors of society, new forms of labor-market distribution have emerged, collectively termed the gig economy (van Zoonen et al., 2024). In fact, attitudes toward the gig economy have been controversial in recent years. On the one hand, the gig economy is regarded as a more effective and sustainable human resources matching model that enables immediate, efficient connections between labor supply and demand, reduces transaction costs, and unlocks the value of the long tail market. On the other hand, the gig economy implies the deprivation of labor rights and interests (such as social security, the minimum wage, and the right to collective bargaining) and the instability of labor conditions (income) (Montgomery and Baglioni, 2021), which may in turn trigger a series of unconventional psychological and behavioral responses (Rosenblat and Stark, 2016; Kim et al., 2022; Deng and Galliers, 2024; van Zoonen et al., 2024). The research object of this study, crowdworkers, is a form of platform labor in the gig economy. Coined by Howe (2006) in Wired magazine, crowdsourcing refers to online groups collaborating to solve problems. Its growth is evident in a global crowdworker base and expanding platforms, like Amazon Mechanical Turk, Clickworker, Microworkers, and ZBJ.com from China. Crowdsourcing platforms provide infrastructure, tools, and rules for interactions between workers, clients, and creators (Kohler, 2018), attracting adoption by tech firms such as Amazon and Google and legacy enterprises (e.g., Walmart, General Electric, and Johnson & Johnson) alike to balance scalability and workforce sustainability (van Zoonen et al., 2024), moving beyond its startup roots.

Despite the increasing integration of crowd work into modern economic systems, research on its human aspects remains limited. Scholars emphasize platform benefits but often overlook addiction fueled by piecework systems and “making-out” games. Crowdsourcing platforms utilize algorithmic governance, a digital-era tool for capital accumulation, to exploit labor value through technology. They combine material rewards with gamified spiritual incentives to frame work as a “game.” This encourages addictive, repetitive engagement and links workers’ self-actualization to capital management. This gamification also justifies algorithmic exploitation by shifting motivation from rational interest-seeking to an intrinsic drive (Hamari, 2013), shaping crowdworkers’ behavior during crowdsourcing tasks (Morschheuser et al., 2017). While recent research has begun examining the overall well-being of gig workers, the causes and consequences of this specific addictive work mindset, especially its connection to key work outcomes, still need urgent investigation.

Innovative behavior is seen as the key for crowdsourcing platforms and their participants, helping them stay competitive and vital. Recent research shows a more complex link between the psychological states of digital workers and their innovative actions. Wood et al. (2019) highlighted a major paradox between external control created by platform algorithmic management and the internal motivation that workers naturally develop. Deng et al. (2022) connected immersive (addiction-like) flow states to creative identity. Sundararajan (2016) further noted that work instability pushes workers to engage in ongoing self-innovation, leading to a form of adaptive innovation. Collectively, these studies raise an important but less explored question: In a crowdsourced work setting, can work addiction be turned into a drive for innovative behavior under certain conditions?

Against this backdrop, we focused on how the incentive mechanisms of crowdwork platforms make crowdworkers addicted and affect their behavioral intentions and innovative behavior. Specifically, the intense intrinsic motivation linked to addictive behavior, the pursuit of flow experience, and the resilience built through problem-solving in unstable environments collectively constitute a distinct psychological state. This state drives laborers to constantly seek newer and more efficient ways to maintain their addictive work cycle. In other words, innovative behavior might be a necessary strategy for addicts to sustain their addictive state. This hypothesis challenges the traditional view that addiction is solely an alienating phenomenon and aims to explore the complexity of psychological and behavioral outcomes within the context of digital labor. Compared with existing studies, the contributions of this paper mainly lie in three areas. Theoretically, it goes beyond exploitation narratives to propose a generative framework, emphasizing the dialectical relationship between negative psychological states and positive behaviors in specific contexts. In terms of mechanism analysis, it introduces two concepts: resilient innovation and crowdworker addictive behavior. The former refers to sustained innovative behaviors that laborers develop under the influence of addiction to cope with instability, while the latter describes a long-term immersive state reinforced by platform mechanisms. Methodologically and practically, it focuses on addictive high performers, advocating mixed research methods to identify their patterns and providing platform design insights (e.g., algorithmic transparency, fair compensation) for sustainable innovation that maintains flow benefits.

## 2 Literature review

### 2.1 Flow theory

Csikszentimihalyi (1975) defined flow as an optimal immersive state characterized by clear goals, immediate feedback, balanced challenge-skill alignment, merged action-awareness, reduced self-consciousness, and distorted time perception. It was later expanded to connect with flourishing and autotelic personalities (Csikszentimihalyi, 1990). The theory has been refined by Nakamura et al. (2001) and applied to digital contexts, such as social media immersion (Zhou et al., 2025) and e-commerce learning (Wu and Tien, 2024), amid growing concerns about “forced flow” and algorithmic exploitation (Roberts and David, 2023), as flow-related immersion is linked to anxiety and depression. In organizations, flow mediates the impact of self-leadership on performance or satisfaction (Stefan and Virga, 2025) and intersects with self-determination theory (SDT).

Flow and addiction, although both involve intense engagement, are fundamentally different: flow is a state of optimal, self-motivated focus driven by internal motivation, a balanced challenge-skill match, and voluntary control, resulting in positive outcomes like a sense of achievement and increased energy (Kuss and Griffiths, 2012). Addiction, by contrast, is a maladaptive compulsion marked by loss of control, tolerance, and withdrawal, rooted in reward system dysregulation and a shift from initial gratification to compulsive relief of distress, with detrimental impacts on long-term well-being (Goldstein and Volkow, 2011; Li et al., 2025).

### 2.2 Self-determination theory

Self-determination theory (SDT), a well-validated framework for explaining individual motivations (Deci and Ryan, 2000; Gagné and Deci, 2005), provides a theoretical basis for the research hypotheses proposed in this study. It classifies motivations into intrinsic (rooted in activity-specific enjoyment) and extrinsic (driven by instrumental goals like rewards or recognition) types (Ryan and Deci, 2000; Wu et al., 2023), both directly shaping laborers’ task engagement and behaviors. SDT’s three intrinsic motivation dimensions further clarify these dynamics: autonomy is defined as perceived control over actions (Harms et al., 2014), and its component self-esteem (Du et al., 2012) may fuel potential addictive factors and innovative experimentation by fostering task ownership. Competence, which refers to mastering skills needed to achieve personal goals (Ryan and Deci, 2000), aligns with the innate drive for competence enhancement (Van den Broeck et al., 2010). Relatedness centered on the need for social connection (Ryan and Deci, 2000). It strengthens workers’ attachment to crowdsourcing platforms (Fedorenko et al., 2017; Pee et al., 2018), thereby increasing their task involvement (which may lead to addiction) and facilitating collaborative innovation.

Drawing on self-determination theory (SDT) as proposed in Peng et al. (2025), algorithmic management is conceptualized as a double-edged sword: its opaque evaluation and punitive mechanisms undermine gig workers’ autonomy and skills, leading to compulsive platform engagement (digital addiction) and eventual burnout; meanwhile, its structured task assignment and real-time feedback may somewhat satisfy psychological needs, reducing some negative impacts of addiction on mental health.

### 2.3 Addictive behavior of crowdworkers

Crowdwork is a highly flexible form of work that offers crowdworkers recognition, gratification, financial rewards and autonomy (Ye and Kankanhalli, 2017), yet its structural traits carry addictive potential of work. Griffiths (2005) emphasizes that addiction emerges from the dynamic interplay of multiple factors. For example, most crowdsourcing platforms adopt a piecework mechanism, which requires continuous task completion for earnings, task competition when there are more workers than tasks, and rewards and satisfaction from succeeding in task acquisition. These factors foster reduced self-regulation and addictive behaviors.

Internet addiction represents the second addictive dimension for crowdworkers. Individuals may be addicted not only to the characteristics of work and its income but also to the medium through which the addictive behavior is conducted. When the Internet is used as a tool to facilitate problematic behavior, individuals do not become addicted to the Internet itself but to specific IT artifacts (Griffiths, 2000; Widyanto and Griffiths, 2006), including crowdsourcing platforms. These platforms’ gamification affordances, such as competitions, social features and successive challenges (Feng et al., 2018), align with internet gaming disorder characteristics (American Psychiatric Association, 2013). They promote a narrowed focus and flow-like immersion to drive sustained participation.

Crowdworkers’ addiction is a unique multi-dimensional construct that combines work and Internet addiction. It fundamentally differs from traditional addiction’s pathological symptoms and non-pathological states like immersion or high engagement due to distinct contextual and psychological markers. Unlike traditional substance or behavioral addictions (e.g., drug dependency, gaming addiction), which are driven by hedonic pleasure, physiological dependence, and detachment from functional roles, crowdworkers’ addiction is primarily motivated by the structural realities of gig work. Its pathological traits arise from survival needs (e.g., fear of missed earnings) rather than intrinsic gratification, with context-specific “withdrawal symptoms” and maintained awareness of harms. It is also distinct from immersion and high work engagement, which promote autonomy, competence, and relatedness. Crowdworkers’ addiction is externally pressured by platform demands or financial insecurity, endures despite burnout or strained relationships, and undermines those core psychological needs. Existing studies have largely focused on how benefit-related factors influence crowdworkers’ willingness to engage with crowdsourcing tasks. However, whether these mechanisms designed to increase participation among crowdsourced workers affect their mental health is ignored. This study fills the gap in research on addictive behaviors in the context of user behavior on crowdsourcing platforms.

## 3 Qualitative study: in-depth interviews and thematic analysis

Given the limited understanding in academia of how addictive behavior and innovative behavior affect crowdworkers, a mixed-methods study was designed based on the research of Glavin and Schieman (2022). A qualitative research design was employed to identify the factors influencing their addiction and exploratorily propose a model of the mechanism behind crowdworkers’ innovative behaviors from the perspective of addiction.

### 3.1 Sampling

To deeply explore the main factors influencing crowdworkers’ behavior, we conducted qualitative interviews with frontline and experienced crowdworkers. Purposeful criterion sampling, which involves selecting participants based on a predefined key criterion, served as the primary selection strategy during the initial phase of the interview. In the subsequent research phase, a dual approach combining convenience and snowball sampling was used to expand the pool of participants strategically. We considered theoretical saturation reached and ceased adding samples. Ultimately, this study recruited 20 crowdworkers from four major platforms (including ZBJ.com, Proginn.com, Upwork, and Amazon Mechanical Turk) who have been involved in crowdsourcing services for the past 2 years Table 1. These participants demonstrated deliberate heterogeneity in industry, education, and background. The interviews typically last 20–30 min and are conducted either face-to-face or via video/audio platforms. With the participants’ consent, the interviews were recorded and transcribed.

**Table 1:** Interviewees profile (*n* = 20).

Demographic items	Frequency	(%)
Gender		
Male	8	40
Female	12	60
Age		
18–25 years old	6	30
26–35 years old	10	50
36–45 years old	3	15
>45 years old	1	5
Education		
College and below	4	20
Undergraduate	11	55
Master and above	5	25
Current occupation		
Teacher	2	10
Student	1	5
Programmer	4	20
Freelancer	5	25
Crowdsourcing worker	8	40

### 3.2 Qualitative interview process

The semi-structured interview protocol was designed to encourage participants to discuss their concerns and experiences related to crowdsourcing work. Initially, a small-scale pilot interview was conducted, and the final questions were determined after modifications. The interview begins with the opportunity that motivated the interviewees to engage in crowdwork (e.g., “What reasons or opportunities prompted you to become a crowdworker?”; “Which crowdsourcing incentive mechanisms or platform designs attract you?”; “What needs does crowdwork meet for you? Or what value does it bring to you?”). Additionally, the interviewees were asked whether the factors that attracted them to participate in crowdwork would make them addicted or lead to innovative behaviors (e.g., “Do these needs or values motivate you to innovate?”; “Will these needs or values motivate you to continue investing in crowdwork?”). Besides these questions, participants were invited to elaborate on their responses, and follow-up questions were posed to them (Kallio et al., 2016).

### 3.3 Qualitative interview results

This study conducted qualitative coding on 20 organized interview records using NVivo 14. To ensure reliability, it was necessary to provide specific training to the three coders on relevant rules, including coding guidelines, general precautions, and explanations of the coding table. The three coders were required to carefully read and repeatedly analyze the 20 collected and organized interview documents word by word, sentence by sentence, and paragraph by paragraph. Initial codes were drawn from key themes in the interview transcripts and refined through three rounds of review to align with the research objectives. Then, during the topic coding process, two independent coders analyzed 25% of the data, achieving an intercoder agreement of 82% (Kappa = 0.79). Remaining discrepancies were resolved through a third coder and joint discussion to finalize coding frames.

#### 3.3.1 Phase 1: open coding

Open coding is a systematic process that involves decomposing, integrating, and converging original materials through the disorganization and reorganization of qualitative data. This method entails extracting verbatim sentences and abstracting concepts from the data, then categorizing them. To minimize bias and preconceived notions, the coding team thoroughly familiarizes itself with the original interview texts, aiming to derive initial concepts directly from respondents’ words whenever possible. The results of open coding are shown in Table 2.

**Table 2:** Results of open coding.

Example quote	Description	Initial coding
E-6 *“I want to improve my professional knowledge and practice ability by taking orders”*	The intention to enhance professional skills through crowdsourcing work	A01 Skill improvement motivation
C-5 *“In the process of communicating with the demand side, I feel that my communication ability has improved”*	Improve customer communication skills and efficiency	A02 Social skills enhancement
K-11 *“Accumulate knowledge that I do not normally have access to”*	Expand knowledge beyond cognitive boundaries	A03 Heterogeneous accumulation of knowledge
G-23 *“If the demand side is satisfied with my work, they will reach out to me again in the future and even refer me to other new clients”*	Customer recognition brings more new opportunities	A04 Reputation diffusion effect
D-17 *“In my opinion, when the need to complete a task is urgent, I will usually feel the pressure and have new ideas and innovative behaviors”*	Turn work stress into innovation	A05 Pressure transformation mechanism
I-18 *“When I really want to do this thing and when my emotions are mobilized, such a scene will make me likely to have more new ideas”*	A highly engaged work state inspires creative solutions	A06 Work immersion drives innovation
H-20 *“I heard the gold sound as soon as I finished my order”*	Behavioral adjustments driven by an emphasis on real-time data feedback	A07 Immediate feedback dependency
B-34 *“The platform’s reward system and leaderboard design also gave me a sense of gamified engagement, and each time I reached a goal, I was motivated to keep going”*	Deep participation by gamified competition	A08 Gamification involvement
D-40 *“This “addiction” comes from both the internal sense of accomplishment and the external design of competition and incentive. This game-like mechanism not only increased the fun of work, but also enhanced my sense of engagement and accomplishment, making the process more enjoyable and fulfilling”*	Positive feedback elicits continued engagement	A09 Achievement addiction mechanism
A-15 *“Seeing my ranking rise or getting more and more positive reviews motivates me to continuously improve my service quality and order efficiency”*	Value recognition through service evaluation	A10 Self-proof drive
C-25 *“I gained recognition by Posting screenshots of difficult orders in industry forums”*	The ongoing interactions formed in the platform’s online community	A11 Virtual community dependency

#### 3.3.2 Phase 2: axial coding

By further classifying the results of open coding into similar categories, the main category is formed by sorting out and establishing logical relations. By sorting out the categories in open coding, four main categories are formed, which are: gamification affordances, skill enhancement, peer reputation, and addiction-driven innovation. The results of axial coding are shown in Table 3.

**Table 3:** Results of axial coding.

Initial coding	Main them	Quote	Conceptualization	Number of participants mentioning
A01 Skill improvement motivation	AA1 Skill enhancement	J-7 “*Every new skill I pick up makes me eager to take on more complex tasks—there’s always something new to learn, and I cannot stop wanting to push my limits further”*	Skill enhancement → Addiction and innovation	17
A02 Social skills enhancement				8
A03 Heterogeneous accumulation of knowledge				3
A04 Reputation diffusion effect	AA2 Peer reputation	L-20 “*That peer and client trust makes me want to keep delivering my best work. I never want to lose that reputation, so I’m always looking for the next task to prove myself*”	Peer reputation → Addiction and innovation	15
A10 Self-proof drive				6
A11 Virtual community dependency				4
A07 Immediate feedback dependency	AA3 Gamification affordances	M-35 “*Instant feedback after each task and the little ‘win’ notifications make it so addictive. Even when I plan to stop for the day, seeing I’m just 10 points away from the next reward makes me squeeze in one more task*”	Gamification affordances → Addiction and innovation	12
A08 Gamification involvement				18
A09 Achievement addiction mechanism				11
A05 Pressure transformation mechanism	AA4 Addiction-driven innovation	F-16 *“I got so hooked on completing tasks quickly that I started creating my own templates and shortcuts to save time. I keep thinking of new ways to streamline the process”*	Addiction → innovation	23
A06 Work immersion drives innovation				9

Based on the above coding, we identify the antecedents of crowdsourced labor addiction from two levels: the external crowdsourced mechanism design and the internal needs of crowdworkers, namely skill enhancement and peer reputation. Through coding, we also found a potential relationship between the addictions of crowdworkers and their innovative behavior. To further explore the causal relationship between these variables, we constructed a research model based on qualitative findings and existing empirical evidence. Subsequently, we verified it through quantitative research.

## 4 Theoretical framework and hypotheses development

### 4.1 Gamification affordances, addiction and innovation behaviors of crowdworkers

Gamification, which originated in the marketing and service sectors, has become a significant trend in human-computer interaction research, especially in the design of incentives (Hamari et al., 2014; Hamari, 2015; Seaborn and Fels, 2015). Crowdsourcing platforms frequently integrate various gamification features, such as point systems, leaderboards, and achievement badges; progress tracking, feedback mechanisms, and virtual rewards; along with narrative elements, virtual environments, team-based challenges, and customizable avatars (Morschheuser et al., 2017). A key goal of gamification is to induce “flow,” a state of deep immersion where users lose self-awareness and feel in control (Csikszentimihalyi, 1990). This optimal experience, highly sought by game developers to maintain user engagement (Andrade et al., 2016), is increasingly applied in crowdsourcing to enhance task focus (Eickhoff et al., 2012; Feng et al., 2018). However, studies link flow experiences to addictive tendencies: Sun et al. (2015) found mobile game addiction correlates with perceived visibility and flow, while Wu and Yang (2013) identified flow as a predictor of addiction via recreation specialization. These insights suggest that gamification’s immersive design, by fostering flow, may inadvertently lead to addictive behaviors. As one of the interviewees remarks:

*“The platform has a section medal wall. If I do 500 singles, I can unlock the ‘industry master’ label. It’s one of the things that makes me brush the mission hall a dozen times a day.”*

Game mechanics applied in non-game settings can enhance innovation by shaping behavior, developing skills, and engaging participants (Steffen et al., 2015). While traditional organizational studies highlight practices like training and motivation for creativity (Okoe et al., 2018; Karadas and Karatepe, 2019; Song et al., 2019), crowdsourcing platforms that lack physical interaction require gamification to increase employee engagement, expertise, and creative work (Cardador et al., 2017; Murawski, 2021). Gamified elements such as leaderboards trigger psychological responses like competence or social comparison (Xi and Hamari, 2019), which align with creativity research linking achievement motivation to innovative behavior (Jing Zhou and Hoever, 2014). Self-determination theory posits that enjoyable tech-driven interactions increase user engagement and motivation for innovative outcomes (Amabile and Pratt, 2016; Suh and Wagner, 2017). Thus, integrating game design into crowdsourcing can leverage these psychological drivers to foster a more innovative workforce. One of the interviewees conveys this saying:

*“Once, to reach the gold level, I thought about applying the usual note-taking classification method to the data annotation, and I did not expect to mark it quickly and accurately.”*

Combining the above arguments, this study proposes:

*H1*: Gamification affordances are positively related to crowdworkers’ addiction (a) and innovation behavior (b).

### 4.2 Skill enhancement, addiction and innovation behaviors of crowdworkers

Self-Determination Theory (SDT) classifies individual motivation into intrinsic and extrinsic motivation and posits that individuals have three basic psychological needs: autonomy, competence, and relatedness (Deci and Ryan, 1985). Crowdsourced digital laborers can better complete tasks by enhancing their skills, which in turn helps them satisfy their competence needs and gain a sense of accomplishment. This sense of accomplishment then strengthens their intrinsic motivation. The enhancement of this intrinsic motivation may lead to work addiction. Combining with the stimulus–response-reinforcement (SRR) framework (Hull, 1943), skill enhancement serves as a rewarding stimulus that reinforces habitual engagement. Rewarding experiences, such as acquiring useful skills, encourage repeated participation, potentially evolving into compulsive dependency as the cycle of stimulus (skill gain) and response (continued use) is reinforced (Belleville, 1964; Pee et al., 2018; Paschke et al., 2024). This process highlights how skill-focused incentives can drive both goal-directed learning and automatic, addictive platform use.

*“To improve my design skills, I continue to receive various design orders on the crowdsourcing platform, from simple posters to complex web interfaces, and every time I complete one order, I feel that I am one step closer to improving my skills.”*

Skill enhancement activates intrinsic motivation by systematically fulfilling three basic psychological needs of individuals, thereby promoting the generation and sustainability of innovative behaviors. Specifically, skill enhancement first strengthens the sense of competence. By acquiring knowledge in new fields or deepening professional expertise, individuals are more likely to receive positive feedback when facing innovation challenges, thereby providing core confidence for innovative behaviors (Stremersch et al., 2022). Secondly, skill enhancement expands the boundaries of autonomy. When individuals possess more diverse skills, they gain greater autonomy in goal setting and method selection during the innovation process, reducing their reliance on external guidance. This sense of autonomous control lowers the tendency to avoid innovation risks and stimulates the willingness to explore unknown fields (Starke and Ludviga, 2025). Furthermore, skill enhancement facilitates the fulfillment of a sense of belonging. With improved skills, individuals can more easily engage in team collaboration, gaining recognition and support from peers through communication. This fosters a collaborative atmosphere of skill complementarity and knowledge sharing, reduces anxiety about trial and error in innovation and provides emotional support for both collective innovation and the implementation of individual ideas (Weng et al., 2022). The synergistic effect of these three elements activates intrinsic motivation, shifting individuals from passively executing tasks to actively exploring innovation, ultimately enabling the transition from skill accumulation to innovative behavior.

*“I took on a project that required 3D modeling. To do it well, I learned a new software by following tutorials. I tried incorporating cartoon rendering techniques from games into the project, and the client said this creative touch made the plan really come to life!”*

The above discussion leads us to postulate the following hypothesis:

*H2*: Skill enhancement is positively related to crowdworkers’ addiction (a) and innovation behavior (b).

### 4.3 Peer reputation, addiction and innovation behaviors of crowdworkers

Social exchange theory suggests individuals pursue social rewards like reputation through interactions (Blau, 1964). On crowdsourcing platforms, reputation systems (e.g., rankings, activity tracking) incentivize contributions by publicly signaling members’ status and competence. Peer reputation serves as a critical social asset, enabling individuals to establish organizational identity and status within communities (Jones et al., 1997; Anderson et al., 2015). This status-seeking motive drives group-oriented behaviors, as members autonomously evaluate social feedback to cultivate their perceived standing. Wood and Lehdonvirta (2023) point out that platform algorithms reduce gig workers’ reputation to quantified ratings, creating persistent recognition anxiety. To avoid reputation loss (such as negative reviews or ranking declines), workers proactively increase their labor input, which drives the formation of an addiction-like persistent behavioral inertia. Research links social identity processes to addictive patterns, particularly when individuals derive self-worth from group validation (e.g., Buckingham et al., 2013; Dingle et al., 2015; Frings and Albery, 2015). In crowdsourcing contexts, contributors earn peer recognition by demonstrating expertise, which reinforces their professional identity. This reputation acquisition becomes a form of extrinsic motivation under self-determination theory (Nov et al., 2010), where unmet psychological needs amplify dependency on digital validation. Studies indicate that individuals compensating for unfulfilled offline needs may develop compulsive platform engagement when deriving status from online achievements (Wong et al., 2015). Consequently, the pursuit of peer reputation creates a cyclical pattern: contributors persistently seek recognition to satisfy identity-related needs, potentially fostering addictive participation behaviors.

*“Every time I see my delivery being liked and ranked in the top 10 by peers, I can't help but refresh the task hall. I just want to maintain this feeling of recognition.”*

Reputation represents the enduring perceptions of an individual that emerge within a wider social environment, reflecting consistent impressions formed through interpersonal interactions and collective observations (Furnham, 1994; Zinko et al., 2012; Djurdjevic et al., 2017). An individual’s reputation influences the perceived image and expected performance results associated with their innovative conduct. In an innovation competition, Deodhar and Gupta (2023) based on a natural experiment conducted on the Kaggle platform, found that the display of peer reputation features significantly enhances the quality of participants’ innovative solutions by stimulating social comparison motivation and competitive awareness. Particularly under high task complexity, reputation pressure motivates individuals to invest more cognitive resources in optimizing their ideas. When individuals have a favorable reputation, research indicates they tend to proactively protect this standing (Baer et al., 2015), engaging in behaviors that enhance their public image while avoiding actions that might diminish it. Once innovative individuals have built a reputation, they are likely driven to uphold their innovative standing.

*“Seeing that the recommended works from my peers on the homepage received high scores, I made a determined effort to try dynamic illustration techniques that I had never used before. After submitting the manuscript, my peers sent me private messages seeking advice and experience. Now, whenever I have free time, I am thinking about how to create something new that others have never tried before!”*

Based on the above discussion, our study expects:

*H3*: Peer reputation is positively related to crowdworkers’ addiction (a) and innovation behavior (b).

### 4.4 Mediating effects of crowdworkers’ addiction

In the previous discussion, this study identified crowdworkers’ addiction to crowdsourcing from two perspectives. On the one hand, crowdsourcing addiction shows the characteristics of technology addiction and Internet addiction. Research within the Media System Dependency framework has shown that technological dependency is inherently goal-directed, characterized by rational, objective-driven behavior (Carillo et al., 2017). This goal orientation refers to the extent to which an individual’s ability to achieve personal or professional aims hinges on engaging with specific technologies. By virtue of this goal-focused nature, dependency theory tends to emphasize the positive outcomes associated with technological use, as individuals strategically leverage tools to meet their objectives. Studies have shown that greater internet use might actually be positively associated with innovativeness and creative achievement (Devine et al., 2022). On the one hand, crowdsourcing presents a few potentially addictive attributes of work. According to COR theory, individuals seek to obtain and safeguard resources that aid in achieving their objectives; yet, actual resource depletion or the perceived risk of loss can trigger stress responses (Hobfoll, 1989). Individual experience addiction means continuous investment of personal resources and the perceived stress of loss. In the existing literature, employees’ perceived challenge stressors could be positively related to their innovativeness by triggering positive reactions (Ohly and Fritz, 2010; Ren and Zhang, 2015; Ding et al., 2019). This study proposes that crowdworker addiction to crowdsourcing platforms sis goal-oriented and arises from the positive stressors related to innovation behavior.

*H4*: Crowdworkers’ addiction is positively related to their innovative behavior.

As mentioned above, this study proposes that gamification affordance, skill enhancement, and peer reputation influence crowdworkers’ addiction and innovation behavior, respectively. The functional attributes of a system elicit psychological states and emotional responses, which in turn shape behavioral outcomes and facilitate value creation processes (Huotari and Hamari, 2017). The extant literature has conceptualized gamification into a few key aspects: (1) the design (gamification affordances), (2) the psychological outcomes of gamification, and (3) the behavioral outcomes of gamification (Huotari and Hamari, 2017). This theoretical model can be used to explain the mediating role of addiction in this study. The psychological outcome, addiction (in this study), further acts as a mediator for the behavioral outcome, innovative behaviors (in this study), and value creation of gamification. Similarly, crowdworker addiction can also play a mediating role between behavioral outcomes (innovation behaviors) and other affordances of crowdsourcing platforms, such as skill enhancement and peer reputation.

*H5(a)*: Gamification affordance has an indirect effect on crowdworkers’ innovation through crowdsourcing addiction.

*H5(b)*: Skill enhancement has an indirect effect on crowdworkers’ innovation through crowdsourcing addiction.

*H5(c)*: Peer reputation has an indirect effect on crowdworkers’ innovation through crowdsourcing addiction.

**Figure 1:**
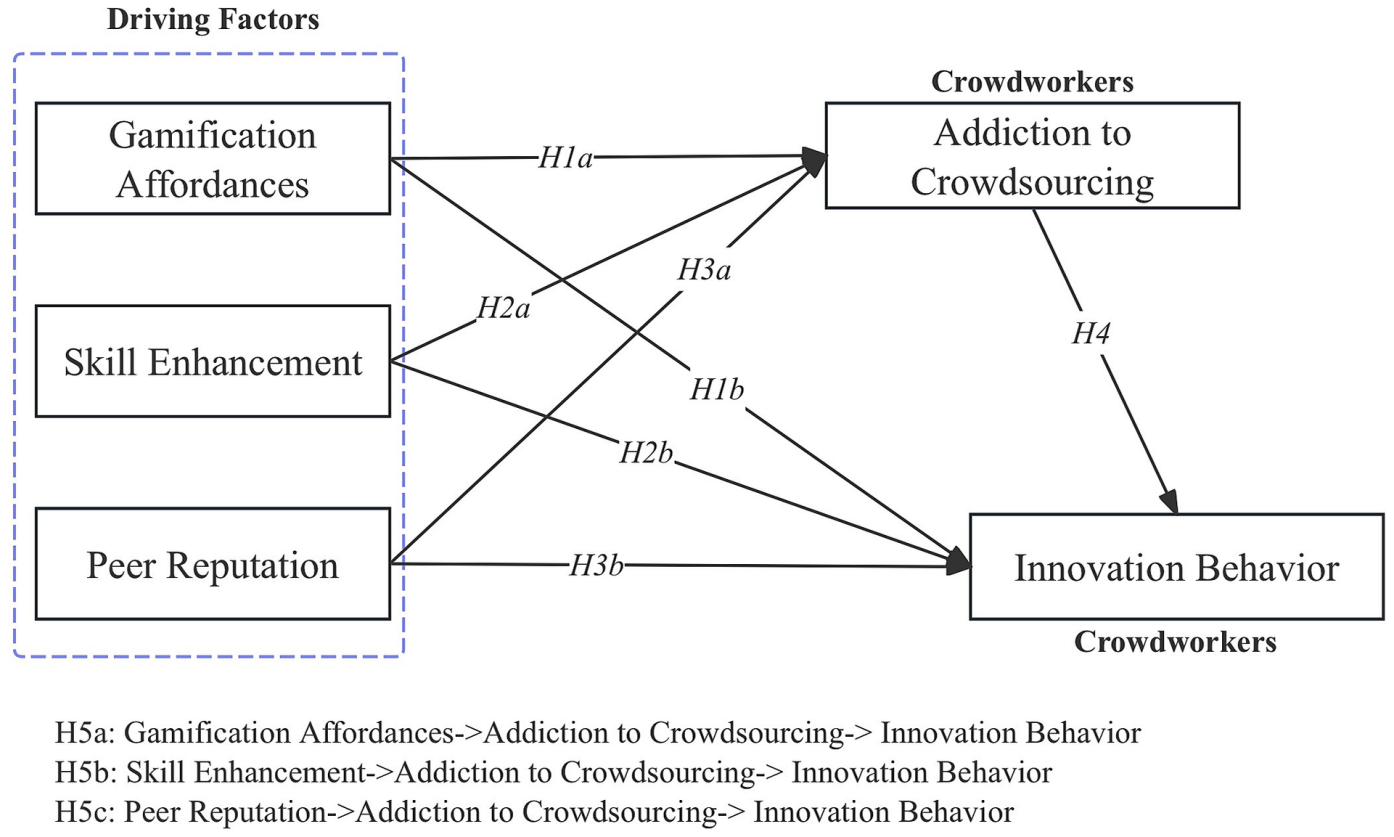
Research model.

Following the above illustrations, I have drawn a figure to present our research model and related hypotheses as shown in Figure 1.

## 5 Quantitative study: a two-wave survey

### 5.1 Data collection

Before formal questionnaire data collection, we conducted a pilot to improve the effectiveness and reliability of the survey tool on epwk.com, a well-known crowdsourcing platform in China. After the pilot phase, we surveyed 112 crowdworkers from epwk.com. Based on the pilot test results, we adjusted the observation items in the scale. During the formal data collection phase, we employed a professional data collection agency to conduct a two-wave survey and gather online digital data. We invited current crowdworkers or those with more than 3 months of crowdsourcing work experience from zhubajie.com and epwk.com to participate in an online survey. It is explained that the questionnaire collects information through an anonymous survey at the beginning. We also pay attention to the security and privacy of the survey object. The results of the survey are used solely for this project and will not be used for any commercial purposes.

The collection interval for the two-wave questionnaire was 1 month. In the first phase, participants were asked to complete measures of gamification affordance, skill enhancement, peer reputation, crowdworkers’ addiction and demographic information. In the second phase, innovation behavior was measured. This method reduces the likelihood that respondents will use their answers to earlier questions to influence their responses to later questions, helping to control for method bias (Podsakoff et al., 2012). The survey yielded 1,036 usable data points. Table 4 summarizes the demographic characteristics of the respondents.

**Table 4:** Respondent profile in quantitative study (*n* = 1,036).

Measure	Item	Frequency	(%)
Gender	Male	526	50.9%
	Female	510	49.0%
Age	18–25	142	13.65%
	26–30	266	25.58%
	31–40	522	50.19%
	41–50	79	7.60%
	51–60	27	2.60%
Education	Junior high school and below	2	0.19%
	High school/vocational school	45	4.33%
	Junior college	129	12.40%
	Undergraduate	773	74.71%
	Graduate and above	87	8.37%
Working industry	Construction Industry	83	7.98%
	Logistics and transportation	83	7.98%
	Education/training	84	8.08%
	IT service	178	17.12%
	Computers/software	174	16.73%
	Wholesale and retail	105	10.48%
	Accommodation and catering industry	66	6.35%
	Financial	61	5.87%
	Real estate	34	3.27%
	Rent/lease	4	0.38%
	Professional services	39	3.75%
	Life services	28	2.69%
	Health care/social security	33	3.17%
	Culture and entertainment	24	2.31%
	Others	40	3.85%

In the SEM analysis, we included demographic (age, gender, education level) and work-related (crowdsourcing platform tenure, weekly work hours, task type) control variables to address potential confounding and enhance model robustness. Platform tenure of crowdworkers may correlate with familiarity-driven efficiency, while task type could directly shape innovation potential. As different task types vary in difficulty, this could influence crowdworkers’ involvement in crowdsourcing (Ye and Kankanhalli, 2017). Their inclusion helped rule out alternative explanations for the observed relationship between addiction and innovation, thereby mitigating omitted-variable bias.

### 5.2 Measurements

This study drew upon mature established measurement tools from prior research in related contexts and adopted them to the specific scope of this study. Survey items were translated from English into Chinese and then subjected to back-translation by several information systems professors fluent in both languages to ensure accuracy and cross-linguistic validity. To enhance the clarity of the measurement items, some volunteers will be approached to observe their understanding of the wording of these items. Next, items with possible confusing descriptions will be refined to express more specific concepts of variables in crowdsourcing. The questionnaire scales employed in the two surveys were developed utilizing a five-point Likert scale anchored from “strongly disagree” to “strongly agree.” The scales developed by this study are attached in Table 5.

**Table 5:** Items of constructs in the proposed model.

Constructs	Items	Source
Gamification affordances	GAM1: I enjoy completing gamified tasks and challenges on the crowdsourcing. Platform	Developed from Eppmann et al. (2018), Feng et al. (2018), Xi and Hamari (2019)
	GAM2: Gamification affordances in the platform sparked my imagination	
	GAM3: While experiencing the gamification affordances in the platform I felt activated	
	GAM4: The gamification of the crowdsourcing platform motivates me to complete more tasks	
Skill enhancement	SKL1: I have opportunities to learn and grow through participating in crowdsourcing tasks	Developed from Ryff (1989), Amabile et al. (1996), Turban and Yan (2016)
	SKL2: I feel that I’ve learned a lot that has made me a more capable person through participating in crowdsourcing tasks	
	SKL3: Through crowdsourcing tasks, I am looking forward to improving my skills in the future	
Peer reputation	REP1: I want other people to find out how good I really can be at my work	Developed from Amabile et al. (1994), Kim et al. (2012)
	REP2: I expect that crowdsourcing platform will increase my reputation record according to my behaviors (e.g., submission, winning the bids)	
	REP3: I am strongly motivated by the recognition I can earn from other people	
Addiction to crowdsourcing	ADD1: I enjoy doing work that is so absorbing that I forget about everything else	Developed from Young (1998), Andreassen et al. (2012)
	ADD2: Working on the crowdsourcing platform has sometimes interfered with other activities in my life	
	ADD3: It is difficult to imagine my work without crowdsourcing platform	
Innovation behaviors	INN1: I often use new working methods, technologies, and instruments	Developed from Scott and Bruce (1994), Ye et al. (2021)
	INN2: I often apply innovative methods to working practice	
	INN3: I develop new ideas from the task feedback	
	INN4: I am an innovative person	

GAM, gamification affordances; SKL, skill enhancement; REP, peer reputation; ADD, Addiction to crowdsourcing; INN, innovation behavior.

### 5.3 Procedural remedies and analysis

To mitigate the risk of Common Method Bias (CMB), the following approaches are used in this study. Firstly, in terms of procedural design, this study conducted a small-scale pilot test before the formal data collection phase. In addition, the questionnaire was divided into two parts and the data collection for these two parts was spaced 1 month apart. During the formal survey, participants were informed that there were no right or wrong answers and that all responses would remain anonymous. Demographic information was collected in the final section of the questionnaire. Secondly, statistical control measures were implemented by Harman’s one-factor test and the variance inflation factor (VIF). Harman’s one-factor test serves as a classical method for assessing CMB and it indicated that no single factor explained over a 50 percent threshold of variance in this study (Podsakoff et al., 2012). The full-collinearity test adopted from the study of Kock (2015). The results indicated that the VIF values ranged from 1.171 to 1.421, which are below the 3.3 thresholds. Consequently, CMB does not pose an issue in the present study.

### 5.4 Evaluation of the measurement model

In this study, PLS-SEM was utilized for model estimation. The measurement model was evaluated using the SmartPLS 4.0 algorithm. We initially assessed the convergent validity associated with latent constructs. Factor loadings, average variance extracted (AVE), composite reliability and Cronbach’s alpha are reported in Supplementary Table S1. The majority of outer loadings exceeded the commendable level of 0.708 (Hair et al., 2019). Albeit these minor discrepancies, the indicator reliability is still bolstered by the AVE score surpassing 0.5 (Sarstedt et al., 2021). Therefore, we can conclude that within our proposed model, the indicators belonging to the same reflexive construct exhibit a high degree of variance among themselves. Thus, the measurement model provides acceptable convergent validity. Subsequently, we assessed the internal consistency reliability linked to latent constructs through composite reliability coefficients (Hair, 2014). Composite reliability values between 0.70 and 0.90 affirmed the high internal consistency reliability of all constructs. Thus, the foundational indicators for each scale demonstrated high reliability for the scales. Finally, as shown in Table 6, the entire results of the HTMT ratio are below the conservative threshold of 0.9 (Henseler et al., 2015), the square roots of AVE were all higher than the correlation coefficients. The analysis results show that the study has established both convergent and discriminant validity.

**Table 6:** Discriminant validity.

Constructs	ADD	GAM	INN	REP	SKL
Fornell-Larcker criterion (1981)					
Addiction to crowdsourcing (ADD)	0.738				
Gamification affordances (GAM)	0.344	0.744			
Innovation behaviors (INN)	0.345	0.570	0.732		
Peer reputation (PEP)	0.257	0.439	0.442	0.751	
Skill enhancement (SKL)	0.293	0.601	0.581	0.571	0.781
Heterotrait-Monotrait ratio					
Addiction to crowdsourcing (ADD)					
Gamification affordances (GAM)	0.407				
Innovation behaviors (INN)	0.434	0.786			
Peer reputation (PEP)	0.331	0.665	0.674		
Skill enhancement (SKL)	0.375	0.846	0.832	0.886	

### 5.5 Evaluation of the structural model

Following the confirmation of measurement model reliability and validity, the analysis proceeded to evaluate the structural model to empirically validate the hypothesized causal pathways. Firstly, all variance inflation factor (VIF) values are clearly below the threshold of 5.0 (Table 7) (Hair, 2014). Thus, collinearity between exogenous constructs does not pose an issue in the structural model. To evaluate the results of the structural model, we evaluated the significance of the path coefficient, R^2^, and the predictive relevance, Q^2^ (Table 7).

**Table 7:** Assessment of structure model.

Hypothesis	**β**	*p*-value	95% BCa-CIs	Result	VIF	*f* ^2^	*R* ^2^	*Q* ^2^
H1a: GAM → ADD	0.250	0.000	[0.188; 0.310]	Supported	1.598	0.045		
H1b: GAM → INN	0.297	0.000	[0.235; 0.360]	Supported	1.670	0.094		
H2a: SKL → ADD	0.086	0.013	[0.021; 0.148]	Supported	1.916	0.004		
H2b: SKL → INN	0.306	0.000	[0.245; 0.368]	Supported	1.925	0.086		
H3a: REP→ADD	0.100	0.005	[0.034; 0.164]	Supported	1.517	0.008		
H3b: REP→INN	0.104	0.002	[0.045; 0.165]	Supported	1.529	0.013		
H4: ADD→INN	0.127	0.000	[0.085; 0.168]	Supported	1.160	0.025		
H5a: GAM → ADD → INN	0.031	0.000	[0.019; 0.046]	Supported				
H5b: SKL → ADD → INN	0.012	0.016	[0.004; 0.022]	Supported				
H5c: REP → ADD → INN	0.016	0.003	[0.008; 0.028]	Supported				
Addiction to crowd sourcing							0.138	0.131
Innovation behavior							0.437	0.415

Bootstrapping based on *n* = 10,000 bootstrap samples. Effects from the control variable are assessed by applying a two-tailed test (PCI 95%). GAM, gamification affordances; SKL, skill enhancement; REP, peer reputation; ADD, Addiction to crowdsourcing; INN, innovation behavior.

**Figure 2:**
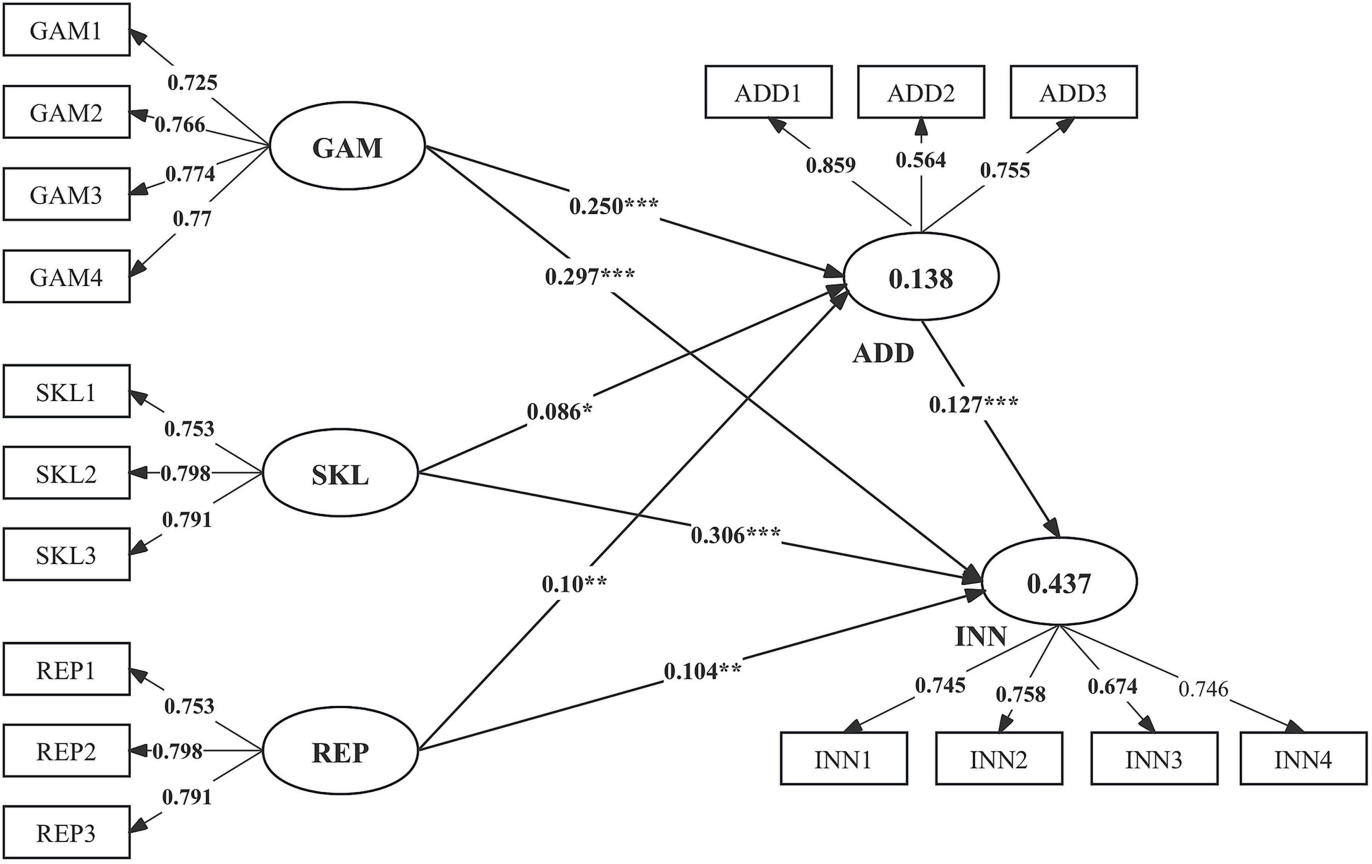
Structural model results. *p < 0.05; **p < 0.01; ***p < 0.001. GAM, gamification affordances; SKL, skill enhancement; REP, peer reputation; ADD, Addiction to crowdsourcing; INN, innovation behavior.

As shown in Figure 2 and Table 7, the structural modeling analysis revealed a significant association between gamification affordances, skill enhancement and peer reputation with addiction to crowdsourcing and innovation behavior. Gamification affordances ( β = 0.250; *p* < 0.001), skill enhancement ( β = 0.086; *p* < 0.05) and peer reputation ( β = 0.100; *p* < 0.01) increase addiction to crowdsourcing, supporting H1a, H2a, and H3a. Moreover, Gamification affordances ( β = 0.297; *p* < 0.001), skill enhancement ( β = 0.306; *p* < 0.001) and peer reputation ( β = 0.104; *p* < 0.01) also positively influence innovation behavior of crowdworkers, supporting H1b, H2b, and H3b. The findings show that addiction to crowdsourcing ( β = 0.127; *p* < 0.001) significantly influences innovation behavior, supporting H4. Further, findings of effect size ( $f^{2}$ ) are presented in Table 7. The other hypothesized paths H1b and H2b had a medium effect. The $Q^{2}$ values for all endogenous constructs were greater than 0, indicating the acceptable predictive relevance of the model.

In addition, the mediation effect of addiction between gamification affordances (β indirect effect = 0.031, *p* < 0.001), skill enhancement (β indirect effect = 0.012, *p* < 0.05), peer reputation (β indirect effect = 0.016, *p* < 0.01), and innovation behavior were found to be significant, indicating partial complementary mediation and supporting H5a, H5b, and H5c.

### 5.6 Reverse causality and endogeneity tests

We employed the Gaussian copula approach (Park and Gupta, 2012; Hult et al., 2018; Liengaard et al., 2025). Prior to conducting the Gaussian copula analysis, we confirmed non-normality via Cramér-von-Mises tests on PLS-derived composite scores for gamification (GAM), skill enhancement (SKL), Peer reputation (REP), and crowdworkers’ addiction (ADD) (all *p*-values < 0.01), which is a key assumption. We modeled these constructs as potentially endogenous, adding copula terms in SmartPLS 4.0 and estimating via bootstrapping.

Gaussian copula results, reported in Table 8, reveal partial endogeneity: only the GCSKL → ADD term is significant (β = −0.225, *p* < 0.01), with a joint Wald test confirming overall relevance (*p* = 0.012). Post-correction (retaining significant terms), most paths remain robust, but SKL → ADD strengthens substantially (β = 0.277, *p* < 0.001 vs. original β = 0.086, *p* < 0.1). Non-significant copula terms were removed to avoid over-specification. Model fit improves marginally (R^2^_ADD = 0.42 vs. original R^2^ = 0.38), with no substantive changes to downstream effects (e.g., SKL → INN, β = 0.367, *p* < 0.001). The results reveal that unobserved factors underestimated the effect of SKL on ADD (from 0.086 to 0.277), rather than inflating spurious links. This correction validates our hypothesis that skill enhancement via platform learning exacerbates crowdworkers’ addiction, unmasking its full intensity amid gig economy pressures.

**Table 8:** Gaussian copula path coefficients.

Path	Original β	*p*-value	Corrected β	*p*-value	GC β (if retained)	*p*-value
GAM → ADD	0.250	***	0.251	***	0.039	ns
GAM → INN	0.297	***	0.297	***	0.129	ns
SKL → ADD	0.086	*	0.277	**	−0.225	**
SKL → INN	0.306	***	0.306	***	−0.062	ns
REP → ADD	0.100	**	0.099	**	0.091	ns
REP → INN	0.104	**	0.104	**	−0.021	ns
ADD → INN	0.127	***	0.127	***	−0.030	ns

Original = without GC; Corrected = with significant GC terms. ***p < 0.001, **p < 0.01, *p < 0.05, ns = not significant. *N* = 1,036. GAM, gamification affordances; SKL, skill enhancement; REP, peer reputation; ADD, Addiction to crowdsourcing; INN, innovation behavior.

We also conducted robustness checks via bidirectional SEM in lavaan (Rosseel, 2012) with the multi-item indicators. Results support our directional hypotheses: forward paths were significant (*p*-values < 0.01 of all forward paths), while reverse paths were non-significant (*p*-values > 0.02 of all reverse paths), indicating no substantial reverse causality. Model fit was acceptable (CFI = 0.881, RMSEA = 0.061, SRMR = 0.076). And OLS regressions with controls (Gender and Age) yielded stable coefficients. Gender and Age were non-significant (*p* > 0.13), suggesting minimal demographic bias. These analyses strengthen the interpretation of our main results by ruling out reverse causality and key confounds, underscoring the robustness of the relationships between GAM, SKL, REP, and ADD.

### 5.7 $\mathrm{PL}S_{\text{predict}}$ for model assessment

In line with the Shmueli et al. (2019) guidelines, this study further assessed the model’s out-of-sample predictive performance. As demonstrated in Table 9, the values of the ${Q^{2}}_{\text{predict}}$ statistic were all greater than 0, indicating predictions of this study model outperform the most naïve benchmark. Given that the skewness values of the prediction errors were lower than |1|, the RMSE values of innovation behavior indicators were adopted. Subsequently, the RMSE values were compared against the naïve linear model (LM) benchmark. The analytical results revealed that all indicators within the PLS-SEM model exhibited lower RMSE values than the LM values, demonstrating superior predictive accuracy.

**Table 9:** $\mathrm{PL}S_{\text{predict}}$ assessment on manifest variables.

Indicators of innovation behaviors	${Q^{2}}_{\text{predict}}$	PLS-SEM RMSE	LM RMSE	PLS-SEM-LM RMSE
Innovation behaviors1	0.232	0.783	0.789	−0.005
Innovation behaviors2	0.234	0.827	0.828	−0.001
Innovation behaviors3	0.211	0.744	0.746	−0.003
Innovation behaviors3	0.207	0.783	0.789	−0.006

RMSE, root mean squared error; PLS, partial least squares; LM, linear regression model. *k* = 7 subgroups, number of repetitions = 10.

## 6 Conclusions and discussions

This study conducted a qualitative analysis of the antecedents of crowdworker addiction and developed a research model based on existing literature and relevant theories. We interviewed 20 Chinese crowdworkers in-depth and performed a coded analysis of the results to build the theoretical model. Subsequently, through a two-wave questionnaire survey, the study revealed how these antecedents influence both the addiction and innovative behavior of crowdworkers, as well as how crowdworker addiction affects their innovative behavior. The results indicate that there are two levels of antecedents related to crowdworker addiction: external incentive mechanisms and internal personal pursuits. These sources of pressure include gamification affordances, skill enhancement, and peer reputation. They further impact the innovative behavior of crowdworkers. Specifically, gamification affordances, skill enhancement, and peer reputation positively influence crowdworker addiction, and these factors also positively affect the innovative behavior of crowdsourcing workers. Additionally, crowdworker addiction has a positive effect on their innovative behavior.

Moreover, the partial complementary mediation of crowdworkers’ addiction between the three driving factors and innovative behavior also exists. This partial mediation underscores a dual pathway. Gamification affordances, skill enhancement, and peer reputation directly influence crowdworkers’ innovation and indirectly through crowdsourcing addiction, as compulsive and sustained platform engagement boosts creative output. This addictive engagement overlaps with adaptive motives, increasing the intensity of goal pursuit while distorting the quality of motivation, shifting it from self-driven engagement to compulsive dependence on platform feedback. Theoretically, this advances digital labor and innovation scholarship by challenging simplified frameworks that view innovation as either purely intrinsic or externally imposed, and by emphasizing the complexity of motivation in gig-economy settings, where adaptive drivers and problematic engagement can coexist to shape creative performance.

While this study finds that addiction has a positive effect on the innovation of crowdsourced workers, an ethical issue of describing addiction as a possible productivity booster still needs to be thought about. Describing addiction as a possible way to boost productivity comes with important ethical risks, mostly because it makes compulsive digital behaviors seem acceptable. Even though high levels of engagement might temporarily increase work output, framing these addiction-related states as desirable hides their unhealthy nature. This hiding makes workers more likely to face problems like burnout, working too much for a long time, or being taken advantage of by the way platforms are designed (Lloyd, 2017). An increasing number of digital workplaces incorporate gamification elements, such as points or rankings, blurring the distinction between flow’s healthy focus and addiction’s compulsive patterns. This mix-up creates productivity traps, in which workers sacrifice their mental well-being to meet performance goals (James, 2021). Making this pattern seem normal not only downplays the official signs of addiction, like losing control or feeling bad when you stop, but also lets widespread exploitation happen. It does this by treating unsustainable work habits as signs of hard work that pays off. These risks match up with new research on digital work. For example, some platforms create feedback loops that trigger short bursts of dopamine, a brain chemical linked to pleasure, rather than the steady release that comes with flow. These loops might inadvertently encourage addictive behaviors, even as the platforms call this intense engagement “top productivity.”

### 6.1 Theoretical contribution

This study employed a mixed-methods approach to conduct content analysis, aiming to identify the factors affecting crowdworkers’ addiction and innovation behavior from the crowdworker perspective. The primary theoretical contributions of this study can be summarized as follows.

First, this study advances the frontiers of crowdsourcing research. A characteristic of emerging disciplines, early crowdsourcing research primarily comprised conceptual works that relied on case-based analyses and descriptive explorations of the phenomenon. Estellés-Arolas and González-Ladrón-de-Guevara (2012) analyzed existing definitions of crowdsourcing and identified over 40 distinct conceptualizations in prior literature. Early scholarly efforts focused on identifying the fundamental components of crowdsourcing as a key research objective to establish conceptual clarity. Albors et al. (2008) developed a crowdsourcing taxonomy, linking it to related concepts like wikis and open-source models while demonstrating its role as an exemplar of an emerging learning network paradigm. Saxton et al. (2013) posited that crowdsourcing represents the convergence of core components, such as the crowd, outsourcing mechanisms, and social web technologies. Many scholars hold that crowdsourcing is an important type of open innovation (Marjanovic et al., 2012; Remneland Wikhamn and Wikhamn, 2013). Thus, crowdsourcing ranks among the most commonly employed terms in open innovation research (Ebner et al., 2009).

Recent research on various aspects of crowdsourcing has focused on the basic elements of crowdsourcing, the factors of the crowdsourcing model, task assignment models, performance and benefits for organizations using the crowdsourcing model, risks of crowdsourcing, motivations of crowdworkers, ethics of users on crowdsourcing platforms, and more. Depending on the perspective adopted, crowdsourcing studies can be roughly classified into three categories: the crowdsourcers’ perspective (those who initiate tasks and offer rewards), the crowdworkers’ perspective (those who respond to advertisements, undertake and submit work, and receive financial compensation), and the crowdsourcing platform or task assignment perspective (those providing an online platform or website where tasks are advertised). These three perspectives offer a holistic view of the crowdsourcing landscape, revealing the complex interplay between task initiators, participants, and the platforms that connect them. This study focuses primarily on the crowdworkers’ perspective and seeks to answer two core questions: What factors affect crowdworkers’ addiction, and how does crowdworkers’ addiction contribute to innovation? By providing empirical evidence and theoretical insights, this study enriches the academic discourse and contributes to the extant literature on this topic.

Second, this study innovatively defines crowdworkers’ addiction through the dual dimensions of work addiction and technology addiction and provides an addiction measurement scale applicable to crowdsourcing contexts. It offers a methodological tool for quantitative research in this field. Crowdworkers’ addiction connects to the concept of “work addiction” in organizational behavior. Still, it transcends the limitations of traditional workplace scenarios, revealing the mechanism of task dependence in non-employment relationships under the crowdsourcing model. Crowdworker addiction also reflects the interactive dependence on platform interfaces, a focus of technology addiction. Crowdsourcing platforms build “behavior-feedback-reinforcement” mechanisms through technical design (such as gamification), leading crowdworkers to form habitual dependence on technical interfaces unconsciously. Unlike traditional addiction research, which emphasizes negative consequences, this study highlights the positive behavioral effects (e.g., innovation) of crowdsourcing labor addiction. It expands the scope of outcome variables in addiction research and provides a new perspective for understanding the duality of addiction in the digital age. Moreover, in line with the COR theory’s premise that individuals are inclined to acquire new resources to achieve loftier goals through judicious resource investment, this study interprets goal-oriented addiction among crowdworkers as a calculated investment of resources. Such investments are made with the aspiration of achieving higher-order objectives, namely innovation.

Third, existing research on employee innovative behavior has largely centered on traditional organizational settings, frequently examining factors such as personality traits, organizational culture, leadership styles, and team dynamics. However, these insights may not fully apply to crowdsourcing contexts, where the absence of fixed hierarchical structures introduces new variables affecting innovation. Unlike conventional organizations, crowdwork platforms rely less on workplace environments or leadership influences and more on the social networks and relational resources of participants. Social networks, which facilitate access to diverse ideas and perspectives, become critical for knowledge recombination and innovative performance (Perry-Smith and Mannucci, 2017). Despite the growing importance of crowdworkers’ social network positions and reflected self-efficacy (Ohlsson, 2011), scholarly exploration of these elements in crowdsourcing remains limited. By shifting focus from traditional organizational determinants to the unique structural and relational features of crowdsourcing, this study expands the theoretical landscape, identifying uncharted factors that shape crowdworkers’ innovative behavior in this emerging collaborative paradigm.

In addition, our finding that crowdsourcing addiction positively predicts innovation aligns with and extends Hamari et al. (2014), who emphasized gamification’s context-dependent effects (not universal) and noted an underexplored gap: the long-term constructive outcomes of gamification-driven over-engagement. Our research shows that gamification-induced crowdsourcing addiction facilitates innovation in gig work, reflecting gamification’s non-binary impact shaped by platform design. Gamified elements (e.g., creative challenges, peer badges) create a feedback loop where addictive engagement channels into innovative problem-solving, advancing Hamari et al.’s framework on motivational affordances. We also extend the analysis of Wood and Lehdonvirta (2023) about recognition struggles in gig work. Our results fill the gap in productive outcomes of overinvestment by identifying crowdsourcing addiction (recognition-driven overinvestment) as a predictor of innovation.

### 6.2 Practical implications

This study adopts a pragmatic approach, offering actionable insights for crowdworkers to leverage the design mechanisms of crowdsourcing platforms to enhance their innovative contributions. Additionally, it provides guidance to firms and platform administrators on fostering crowdworkers’ innovation in the crowdsourcing context. Specifically, this study contributes to practice in three key ways.

First, it empowers crowdworkers to harness the motivational and addictive elements of crowdsourcing platforms to drive innovation. Crowdworkers often engage in crowdsourcing to gain recognition, showcase their skills, expand professional networks, and earn rewards. The design mechanisms of these platforms, such as gamification, instant feedback, and reward systems, can foster task immersion and habitual participation, which this study identifies as potential drivers of innovative behavior. By understanding how these mechanisms trigger engagement, crowdworkers can strategically channel their task-oriented focus into creative problem-solving and high-quality outputs. For instance, they can leverage the motivational pull of virtual badges or leaderboards to sustain engagement while prioritizing innovative contributions over mere task completion. This perspective encourages crowdworkers to transform platform-induced addiction into a productive force for enhancing their personal value and creative performance.

Second, it provides actionable recommendations for firms and crowdsourcing platform administrators to promote crowdworkers’ innovative behavior. The findings highlight how enhancing perceptions of gamification enjoyment, skill development, and peer reputation can stimulate innovation. To this end, platforms should develop sustainable business models and monetization strategies that incentivize innovative contributions, such as revising revenue-sharing mechanisms to reward high-quality, creative outputs. Additionally, platforms can emphasize the engaging and enjoyable aspects of participation to deepen involvement. For example, they could create a webpage for sharing experiences where crowdworkers highlight positive, inspiring crowdsourcing experiences. Platforms could also implement virtual gamification reward systems, such as badges or points, to enhance enjoyment. Furthermore, platforms should promote the autonomy of crowdworkers to realize the value of their skills by enabling flexible task engagement, for instance, through targeted campaigns that emphasize self-directed work. Finally, platforms can underscore opportunities for skill development, positioning crowdsourcing as a pathway to build and refine expertise in specific domains.

Third, by identifying key factors in the development of crowdsourcing models and proposing targeted operational enhancements, this study contributes to the evolution of the crowdsourcing ecosystem. These advancements promote an open, collaborative, and mutually beneficial utilization of social resources, thereby improving production efficiency and supporting sustainable development. As the sharing economy continues to disrupt traditional employment paradigms, society is transitioning toward an era of open crowdsourcing, where organizational boundaries blur and individual empowerment flourishes. The independent working model is becoming increasingly mainstream, enabling efficient connections between talent and platforms. In an internet-driven, fully open labor market, individuals engage in crowdsourcing, exchange labor contracts, and participate in service exchanges, thereby optimizing the utilization of social resources and enhancing overall production efficiency.

The current study emphasizes the positive effects of crowdsourcing platform addiction mechanisms on workers’ innovation performance, highlighting how increased engagement and task immersion can boost creative output. However, it is crucial not to ignore the potential negative impacts of such an unhealthy psychological state on crowdsourcing workers’ overall well-being. The compulsive involvement, blurred boundaries between work and personal life, and chronic psychological stress linked to platform addiction may gradually weaken workers’ mental resilience, resulting in burnout, emotional exhaustion, and even long-term psychosocial issues. From a practical perspective, this underscores the need for a balanced approach among key stakeholders: crowdsourcing platforms should improve algorithm designs to minimize triggers that induce addiction. Based on the empirical results of Hajiheydari and Delgosha (2024), it is recommended that the platform introduce an “algorithm stress index” (such as task allocation frequency and system response delay) and automatically trigger digital disconnection reminders for high-risk users to break the vicious cycle of algorithm dependence and psychological exhaustion. Employers and project initiators should prioritize fair task allocation and reasonable workload arrangements, avoiding over-reliance on workers’ addictive tendencies to drive innovation. Policymakers, meanwhile, could develop guidelines for platform governance that balance innovation incentives with labor protection, ensuring crowdsourcing workers’ rights to psychological health are not compromised for creative gains. Ultimately, fostering sustainable innovation in crowdsourcing requires reconciling the short-term creative benefits of platform engagement with the long-term well-being of workers, as the latter constitutes the fundamental cornerstone of sustained creative productivity.

### 6.3 Limitations and future research directions

This study, which utilizes cross-platform samples to investigate crowdworkers’ addiction and innovation, has certain limitations. First, the platforms sampled may not cover all types of crowdsourcing models, such as contest-based, microtask, or domain-specific platforms, which could limit the generalizability of the results. Future research could explore a wider variety of platform types, examine their impacts on addiction dimensions, and include platform type as a moderating factor. Second, the study does not extensively analyze platform-specific addiction dynamics in specialized fields. Case studies of such platforms could enhance addiction theory and help develop customized governance strategies for different platforms within the gig economy.

Furthermore, the limitations in the research design stem from the two-wave approach, which offers limited support for establishing causality but allows for preliminary exploration of the temporal relationships between variables. Additionally, the collection of demographic data can be improved by including factors such as job type, working hours, and income levels. Moreover, the measurement of key constructs (e.g., innovative behavior) mainly depends on self-reported data, without external evaluations or objective metrics (e.g., platform-recorded task-optimization outcomes, peer validation of innovative practices) to cross-verify findings, which may introduce bias. First, social desirability bias may be present. Participants might overstate their innovative behaviors to align with socially positive perceptions of being innovative, leading to inflated response values. Second, recall bias may arise, as crowdworkers may struggle to accurately recall the frequency, depth, or specific details of Second, recall bias may occur because crowdworkers might find it difficult to accurately recall the frequency, depth, or specific details of their innovative practices (e.g., tool optimization, process improvements) over a certain period, especially for occasional or less noticeable acts. Third, subjective cognitive bias may emerge. People might have different interpretations of innovative behavior, with some viewing minor changes as innovation and others setting higher standards, resulting in inconsistent response criteria. These limitations should be considered when interpreting the findings and replicating the study design in future research.

Future research will address the identified limitations through targeted improvements, focusing on enhancing causal inference rigor and measurement reliability. Firstly, adopt a multi-wave longitudinal design with extended time intervals (e.g., 3–4 waves over 6–12 months) and cross-lagged panel analysis. This design will better capture temporal sequencing between variables, strengthening causal identification beyond the two-wave framework’s limited explanatory power. Secondly, integrate objective metrics and external evaluations to complement self-reported data. Specifically, incorporate platform-recorded indicators (e.g., task efficiency improvements, innovative tool adoption rates, client-approved process optimizations) and peer and client assessment surveys to cross-validate constructs such as innovative behavior, thereby reducing self-report bias. And employ a mixed-methods approach combining quantitative surveys with qualitative follow-up interviews or focus groups. This will deepen understanding of the psychological mechanisms underlying crowdworkers’ addiction and innovative behavior, while triangulating findings to enhance validity. Thirdly, control for additional confounding variables (e.g., task complexity, platform algorithm type, individual differences in self-regulation) and use advanced statistical techniques (e.g., propensity score matching, instrumental variable analysis) to mitigate endogeneity further and strengthen the robustness of causal inferences.

## Data Availability

The original contributions presented in the study are included in the article/Supplementary material, further inquiries can be directed to the corresponding author.
