# Supplementary material for: How addiction fuels innovation: a mixed-methods study on the psychological trade-offs of digital labor in gig economy
Source: Front Psychol. 2025 Dec 16;16:1701187. doi: 10.3389/fpsyg.2025.1701187 (PMC12747959; doi:10.3389/fpsyg.2025.1701187)
Supplement: Supplementary file 1 [file Table_1.pdf]

**Supplementary Table**

**Table S1.** Measurement and operationalization

| Constructs | Items | VIF | Loading | Composite reliability | AVE |
|---|---|---|---|---|---|
| *Gamification affordances* Developed from Eppmann et al. (2018); Feng et al. (2018); Xi and Hamari (2019) | *To what extent do you agree the description when you are facing the following situations?* | | | 0.832 | 0.553 |
| | GAM1: I enjoy completing gamified tasks and challenges on the crowdsourcing. platform. | 1.360 | 0.725 | | |
| | GAM2: Gamification affordances in the platform sparked my imagination. | 1.364 | 0.766 | | |
| | GAM3: While experiencing the gamification affordances in the platform I felt activated. | 1.418 | 0.774 | | |
| | GAM4: The gamification of the crowdsourcing platform motivates me to complete more tasks | 1.364 | 0.707 | | |
| *Skill enhancement* Developed from Ryff (1989); Amabile et al. (1996); Turban and Yan (2016) | *To what extent do you agree the description when you are facing the following situations?* | | | 0.824 | 0.610 |
| | SKL1: I have opportunities to learn and grow through participating in crowdsourcing tasks. | 1.297 | 0.753 | | |
| | SKL2: I feel that I've learned a lot that has made me a more capable person through participating in crowdsourcing tasks. | 1.329 | 0.798 | | |
| | SKL3: Through crowdsourcing tasks, I am looking forward to improving my skills in the future. | 1.345 | 0.791 | | |
| *Peer reputation* Developed from Amabile et al. (1994); HW Kim et al. (2012) | *To what extent do you agree the description when you are facing the following situations?* | | | 0.794 | 0.563 |
| | REP1: I want other people to find out how good I really can be at my work. | 1.283 | 0.789 | | |
| | REP2: I expect that crowdsourcing platform will increase my reputation record according to my behaviors (e.g., submission, winning the bids). | 1.139 | 0.660 | | |
| | REP3: I am strongly motivated by the recognition I can earn from other people. | 1.286 | 0.795 | | |
| *Addiction to crowdsourcing* Developed from Young (1998); Andreassen et al. (2012) | *To what extent do you agree the description when you are facing the following situations?* | | | 0.776 | 0.542 |
| | ADD1: I enjoy doing work that is so absorbing that I forget about everything else. | 1.171 | 0.859 | | |
| | ADD2: Working on the crowdsourcing platform has sometimes interfered with other activities in my life. | 1.303 | 0.564 | | |

| | | | | | |
|---|---|---|---|---|---|
| | ADD3: It is difficult to imagine my work without crowdsourcing platform. | 1.325 | 0.755 | | |
| *Innovation behaviors* Developed from Scott and Bruce (1994); P. H. Ye et al. (2021) | *To what extent do you agree the description when you are facing the following situations?* | | | 0.821 | 0.535 |
| | INN1: I often use new working methods, technologies, and instruments. | 1.334 | 0.745 | | |
| | INN2: I often apply innovative methods to working practice. | 1.421 | 0.758 | | |
| | INN3: I develop new ideas from the task feedback. | 1.219 | 0.674 | | |
| | INN4: I am an innovative person. | 1.405 | 0.746 | | |

Notes: GAM, gamification affordances; SKL, skill enhancement; REP, peer reputation; ADD, Addiction to crowdsourcing; INN, innovation behavior.

**Table S2** Respondents' background

| Measure | Item | Frequency | (%) | Measure | Item | Frequency | (%) |
|---|---|---|---|---|---|---|---|
| Gender | Male | 526 | 50.9% | Province/ Municipality | Guangdong | 154 | 14.9% |
| | Female | 510 | 49.0% | | Jiangsu | 83 | 8.0% |
| Age | 18-25 | 142 | 13.65% | | Shandong | 66 | 6.4% |
| | 26-30 | 266 | 25.58% | | Henan | 61 | 5.9% |
| | 31-40 | 522 | 50.19% | | Sichuan | 59 | 5.7% |
| | 41-50 | 79 | 7.6% | | Hebei | 58 | 5.6% |
| | 51-60 | 27 | 2.6% | | Shanghai | 57 | 5.5% |
| Education | Junior high school and below | 2 | 0.19% | | Hunan | 48 | 4.6% |
| | High school/ vocational school | 45 | 4.33% | | Zhejiang | 46 | 4.4% |
| | Junior college | 129 | 12.40% | | Hubei | 44 | 4.2% |
| | Undergraduate | 773 | 74.71% | | Beijing | 42 | 4.1% |
| | Graduate and above | 87 | 8.37% | | Jiangxi | 34 | 3.3% |
| Working industry | Construction Industry | 83 | 7.98% | | Anhui | 28 | 2.7% |
| | Logistics and transportation | 83 | 7.98% | | Guangxi | 28 | 2.7% |
| | Education/Traini ng | 84 | 8.08% | | Liaoning | 26 | 2.5% |
| | IT service | 178 | 17.12% | | Heilongjiang | 26 | 2.5% |
| | Computers/Softw are | 174 | 16.73% | | Fujian | 26 | 2.5% |
| | Wholesale and | 105 | 10.48% | | Yunnan | 23 | 2.2% |

| | | | | | |
|---|---|---|---|---|---|
| Retail Accommodation and catering industry | 66 | 6.35% | Chongqing | 22 | 2.1% |
| Financial | 61 | 5.87% | Shanxi | 22 | 2.1% |
| Real Estate | 34 | 3.27% | Tianjin | 17 | 1.6% |
| Rent/Lease | 4 | 0.38% | Guizhou | 15 | 1.4% |
| Professional services | 39 | 3.75% | Shaanxi | 14 | 1.4% |
| Life services | 28 | 2.69% | Hainan | 11 | 1.1% |
| Health Care/Social Security | 33 | 3.17% | Jilin | 9 | 0.9% |
| Culture and Entertainment | 24 | 2.31% | Xinjiang | 9 | 0.9% |
| Others | 40 | 3.85% | Inner Mongolia | 8 | 0.8% |
